# Charge extraction via graded doping of hole transport layers gives highly luminescent and stable metal halide perovskite devices

**DOI:** 10.1126/sciadv.aav2012

**Published:** 2019-02-15

**Authors:** Mojtaba Abdi-Jalebi, M. Ibrahim Dar, Satyaprasad P. Senanayak, Aditya Sadhanala, Zahra Andaji-Garmaroudi, Luis M. Pazos-Outón, Johannes M. Richter, Andrew J. Pearson, Henning Sirringhaus, Michael Grätzel, Richard H. Friend

**Affiliations:** 1 Cavendish Laboratory, Department of Physics, University of Cambridge, JJ Thomson Avenue, Cambridge CB3 0HE, UK.; 2 Laboratory of Photonics and Interfaces, Institute of Chemical Sciences and Engineering, École Polytechnique Fédérale de Lausanne, Lausanne CH-1015, Switzerland.; 3 Clarendon Laboratory, Department of Physics, University of Oxford, Parks Road, Oxford OX1 3PU, UK.; 4 Department of Electrical Engineering and Computer Sciences, University of California, Berkeley, CA 94720, USA.

## Abstract

One source of instability in perovskite solar cells (PSCs) is interfacial defects, particularly those that exist between the perovskite and the hole transport layer (HTL). We demonstrate that thermally evaporated dopant-free tetracene (120 nm) on top of the perovskite layer, capped with a lithium-doped Spiro-OMeTAD layer (200 nm) and top gold electrode, offers an excellent hole-extracting stack with minimal interfacial defect levels. For a perovskite layer interfaced between these graded HTLs and a mesoporous TiO_2_ electron-extracting layer, its photoluminescence yield reaches 15% compared to 5% for the perovskite layer interfaced between TiO_2_ and Spiro-OMeTAD alone. For PSCs with graded HTL structure, we demonstrate efficiency of up to 21.6% and an extended power output of over 550 hours of continuous illumination at AM1.5G, retaining more than 90% of the initial performance and thus validating our approach. Our findings represent a breakthrough in the construction of stable PSCs with minimized nonradiative losses.

## Teaser

Charge extraction via graded hole transport layers boosts luminescence, lifetime, and efficiency of perovskite solar cells.

## INTRODUCTION

Metal halide perovskites have attracted tremendous interest for optoelectronic applications, particularly for solar cells where drastic improvements in power conversion efficiency (PCE) have moved them closer to operating at their theoretical limits (*1*). Nevertheless, the long-term stability of perovskite solar cells (PSCs) remains a pressing challenge that hinders their commercialization (*2*), and substantial nonradiative losses in perovskite devices, mainly originating from interfacial defects, prohibit them from reaching their full potential (*3*). An effective way to remove these defects apart from a chemical modification of perovskite (*4*) is to introduce passivation treatments and interlayers between the perovskite and the electron transport layer (ETL)/hole transport layer (HTL) (*5*, *6*). It has been intensively shown that treating mesoporous TiO_2_, arguably the most common ETL in PSCs, reduces electron trap state density and enables faster electron transport (*7*–*9*). The interface between perovskite and the HTL [e.g., doped 2,2′,7,7′-tetrakis(*N*,*N*-di-*p*-methoxyphenyl-amine)9,9′-spirobifluorene, denoted as Spiro-OMETAD] is equally crucial, and to obtain maximum PCE, this needs to be enhanced simultaneously (e.g., to achieve faster hole transport). We and others have recently shown that p-type contact is the main origin for the quenching of photoluminescence (PL) in perovskite devices and that passivation interlayers can significantly enhance the PL quantum efficiency (PLQE) in the complete device architecture (*10*–*12*). However, many of these passivation approaches can potentially introduce unwanted properties that limit the long-term stability of PSCs (*13*).

An efficient strategy to accelerate charge transport through the layers of an optoelectronic device is to use a graded bandgap concept (*14*). For PSCs, this has been shown through the stacking of two different perovskite layers to obtain a graded bandgap that improves the output photocurrent significantly (*15*). Since operational stability is also a prerequisite for PSC commercialization, it is important that any interlayers and treatments introduced to the PSC do not reduce its lifetime (*16*). Sources of instability in PSCs originate not only from their individual layers [e.g., perovskite layer (*17*) and charge-transporting layers (*18*)] but also from their interfacial regions (*19*). The stability of PSCs has improved significantly through changes in the perovskite composition (*20*) [e.g., doping with monovalent cations (*12*, *21*, *22*)] and substitution of organic HTLs for inorganic counterparts (*23*, *24*). However, the overall device stability is still far from the industrial standard where the most prominent source of instabilities arises from the HTL electrode (e.g., Spiro-OMeTAD), for example, the migration of dopants (*25*) (e.g., Li) and metal from the PSC electrode, particularly at elevated temperatures (*26*). Therefore, development of HTLs with clean interfaces and long-term device stability is still one of the major challenges.

In this study, we demonstrate a dopant-free p-type electrode via thermal evaporation of tetracene on top of the perovskite active layer. Tetracene is a molecular organic semiconductor and the four-ringed member of the acene series; common applications of this material include organic field-effect transistors (*27*) and organic light-emitting diodes (*28*). As an efficient singlet fission material, tetracene also shows potential for energy down-conversion applications (*29*). However, given its location behind the perovskite layer in our PSCs and the mismatch in energy levels that enable energy transfer, we expect that its ability to absorb light and contribute to the PSC photocurrent will be limited. On the other hand, tetracene as a wide-bandgap semiconductor with a favorable highest occupied molecular orbital (HOMO) level at −5.3 eV (*30*) is predicted to be the most suitable HTL for PSCs as this level is close in energy, with the valence band of the perovskites considered to be near −5.4 eV (*31*). In contrast, the HOMO levels for other acenes such as anthracene and pentacene are much deeper (−5.7 eV) and higher (−5.0 eV), respectively. To date, this work is the first report that uses tetracene as an HTL in PSCs. As expected, the efficiency of PSCs using tetracene alone as an HTL is low because of poor ohmic contact between this organic semiconductor and the metal electrode, a result that is consistent with related work on organic field-effect transistors (*32*, *33*). To obtain highly efficient and barrier-free hole extraction to the top electrode (e.g., gold), we show that it is advantageous to deposit a capping layer of Spiro-OMeTAD to form a graded HTL that accelerates the injection of holes from perovskite to the external circuit in PSCs. The efficient hole extraction using this graded doping profile is achieved by providing clean interfaces with perovskite active layer and effective ohmic contact with the metal electrode.

## RESULTS

**Fig. 1:**
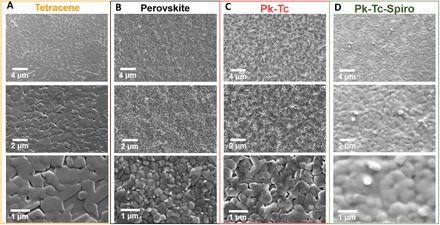
Scanning electron microscopy images. Top-view scanning electron microscopy of (**A**) tetracene (Tc) single layer, (**B**) perovskite (Pk), (**C**) perovskite-tetracene (Pk-Tc), and (**D**) perovskite-tetracene-Spiro (Pk-Tc-Spiro) films at three different magnifications.

In this study, we use the state-of-the-art rubidium-passivated multiple cation–based perovskite, Cs_0.06_FA_0.79_MA_0.15_Pb(I_0.85_ Br_0.15_)_3_, where MA refers to methylammonium (CH_3_NH_3_) and FA refers to formamidinium [CH(NH_2_)_2_], by mixing the precursor solution with rubidium iodide (RbI) stock solution. Notably, this composition shows one of the lowest nonradiative losses in PSCs with reasonable device stability (*12*). We use fluorinated tin oxide (FTO)/compact TiO_2_/mesoporous TiO_2_/perovskite/Spiro-OMeTAD/Au as the standard device architecture (hereafter denoted as Spiro) and an equivalent architecture with tetracene evaporated onto the perovskite layer with a capping layer of Spiro. In Fig. 1, we show the surface morphologies of different layers using a top-view scanning electron microscope (SEM) at different magnifications. Whereas both tetracene and perovskite form compact polycrystalline films containing micrometer-sized grains (Fig. 1, A to C), Spiro adopts a conformal coating (Fig. 1D), ensuring total surface coverage of the combined HTLs. To rule out the effect of Spiro deposition on the thickness of tetracene, we spin-casted chlorobenzene, the solvent used for Spiro on perovskite-tetracene films. We observe no change in the film thickness of tetracene, but the surface morphology of the tetracene particles changed marginally (fig. S1). The cross-sectional scanning electron microscopy of perovskite-tetracene-Spiro thin films further confirms the presence of a conformal tetracene layer (fig. S1) sandwiched between perovskite and Spiro layers.

**Fig. 2:**
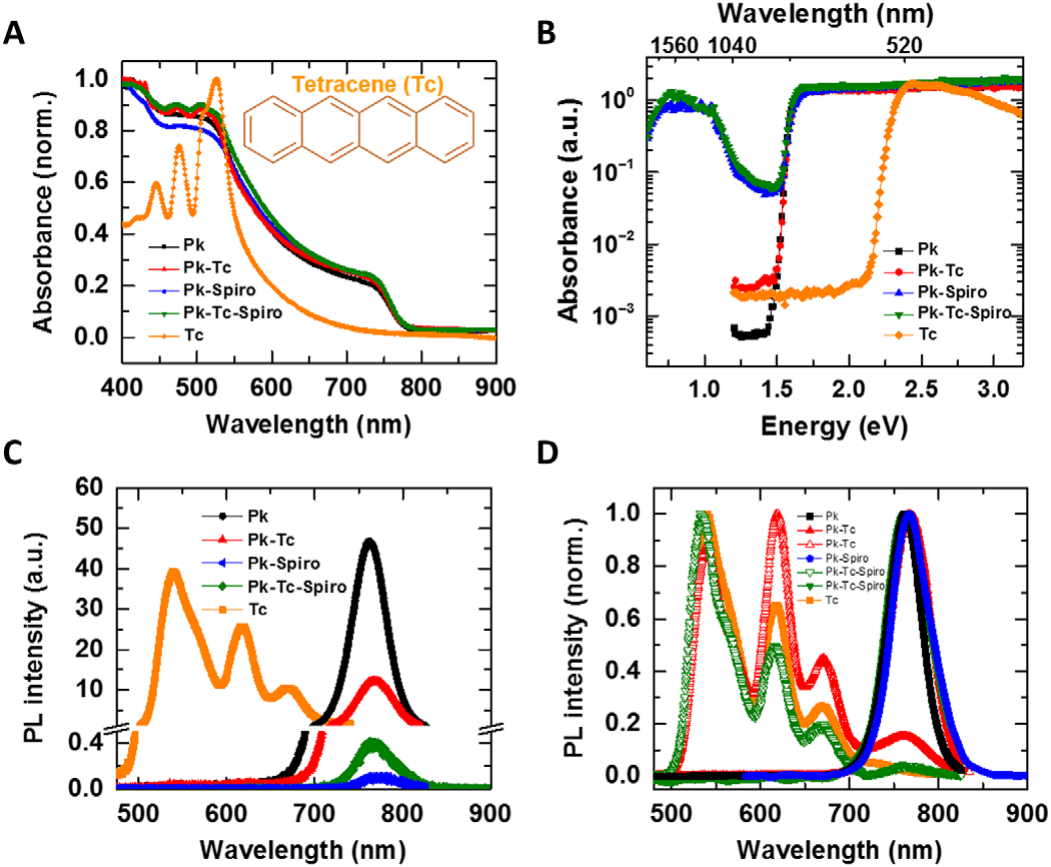
Optical absorption and emission properties. (**A**) Ultraviolet-visible absorption spectra, (**B**) PDS, (**C**) PL, and (**D**) normalized PL spectra, excited using continuous wavelength laser at 407 nm, of the single, double, and triple layers of perovskite, Spiro, and tetracene. The front (HTL side) and back (perovskite side) PL excitations were shown as open and closed symbols, respectively. The break in (C) is from 0.5 to 1. a.u., arbitrary units.

In Fig. 2A, we show the normalized ultraviolet-visible absorption spectra of tetracene and perovskite in different configurations. The three absorption peaks of tetracene that originate from its vibronic structure remain present in the perovskite-tetracene and perovskite-tetracene-Spiro films. Furthermore, higher absorption of the perovskite interfaced with tetracene and combined HTLs above the bandgap can be attributed to the higher light out-coupling in these films. In Fig. 2B, we present photothermal deflection optical absorption spectroscopy (PDS) data that provide insight into optical bandgap, subbandgap states, and energetic disorder in each sample. As expected, the bandgap of perovskite and tetracene appears at 1.59 and 2.31 eV, respectively, and there is no change in the bandgap of perovskite upon the introduction of other layers. Furthermore, we observe a broad peak at low energies for the samples containing a Spiro layer that is attributed to oxidation of Spiro as it is apparent from PDS spectra of an undoped Spiro single layer and its bilayer with perovskite (fig. S2A). This low broad peak becomes more pronounced in the Spiro layer containing dopants such as lithium- and cobalt-based additives, confirming efficient oxidization of Spiro (fig. S2A) (*34*).

PDS offers a bulk-sensitive way of probing subbandgap tail states of the excitonic joint density of states that can be used to estimate the energetic disorder in a semiconductor. The exponential decay of the absorption below the bandgap with a characteristic energy, the Urbach energy (*E*_u_), has been widely used to study perovskite materials. The Urbach energy is given by *A* ∼ exp(*E/E*_u_), where *A* is the absorbance and *E* is the excitation energy in electron volts. The Urbach energy of tetracene (i.e., ∼26 meV) is significantly lower compared to Spiro-OMeTAD (*E*_u_ ∼ 117 meV), indicating a significantly lower density of traps and energetic disorder in the former semiconductor (fig. S2B). This difference in *E_u_* is also reflected in the Urbach energy of the perovskite film interfaced with these HTLs reaching ∼13 meV for perovskite-tetracene and ∼20 meV for perovskite-Spiro, confirming a cleaner interface between perovskite and tetracene.

In Fig. 2C, we show the corresponding steady-state PL spectra of the abovementioned films. PL from tetracene in the perovskite-tetracene sample is not detected when exciting the perovskite layer first, an observation that can be explained by the large optical density of the perovskite at the excitation wavelength. However, if we illuminate the samples from the tetracene side, its luminescence can be measured (Fig. 2D). To study whether the singlet exciton fission process is occurring between tetracene and perovskite, we performed magnetic PL measurements where we measure the changes in the PL upon applying a magnetic field and a continuous laser excitation of 405 nm. It is well known that the transfer of either singlets or triplets from the triplet sensitizer (e.g., tetracene) to the low bandgap semiconductor induces magnetic field modulation on the PL from the latter material (*35*). In fig. S2 (C and D), we show the magnetic field responses for tetracene PL peak at 530 nm and perovskite PL peak at 760 nm in the bilayer of perovskite and thermally evaporated tetracene. As expected, the magnetic field response of singlets in tetracene is about 30% for single layers, while we observed no substantial magnetic response for the perovskite peak. This confirms that there is no energy transfer from tetracene to the perovskite because of the notable mismatch between the triplet energy level of tetracene (e.g., 1.3 eV) and the perovskite bandgap (e.g., 1.6 eV).

In Fig. 2C, as expected, the PL of perovskite at 780 nm is quenched significantly when the material is in contact with the charge-extracting layers, both Spiro and tetracene. In fig. S2E, we show the PL spectra measured at time zero using the pulsed laser for the tetracene and tetracene-Spiro films where the huge quench in the PL represents the efficient hole transfer between tetracene and Spiro.

**Fig. 3:**
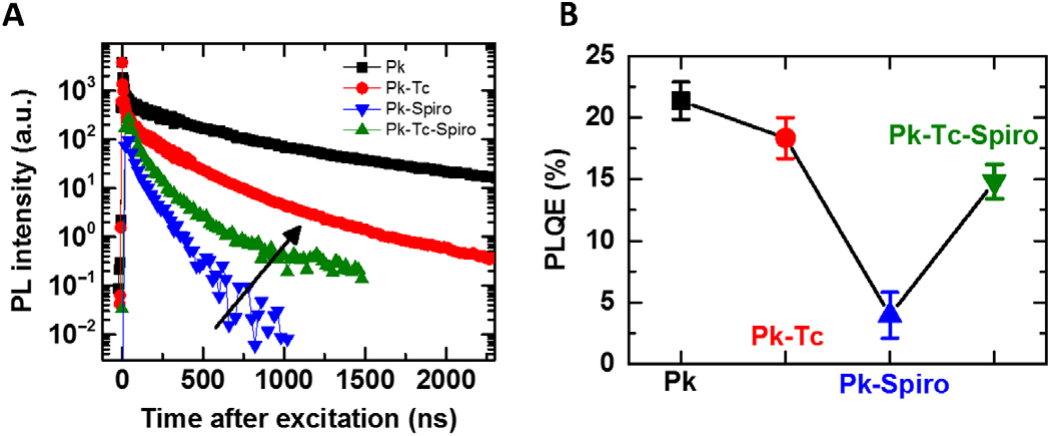
Luminescence properties of the perovskite when interfaced with different p-type contacts. (**A**) TRPL decays of the films with excitation at 400 nm and a pulse fluence of 0.05 μJ cm^−2^ (~1.5 × 10^15^ cm^3^, equivalent to about 1 sun). (**B**) External PLQE of the perovskite interfacing with HTLs in different configurations measured under illumination with a 532-nm laser at an excitation intensity (60 mW cm^−2^) that is equivalent to 1 sun.

A solar cell’s external luminescence efficiency is one important factor that must be maximized to ensure device operation close to its theoretical limit. The p-type electrode is the main source of luminescence quenching in the perovskite device architectures (*10*), and therefore, providing a clean interface between perovskite and HTL is essential to maximize the external luminescence in PSCs. In Fig. 3, we show time-resolved PL (TRPL) decays and PLQE for the perovskite when interfaced with different HTL configurations where the integral of the PL decays in Fig. 3A that defines the radiative efficiency of the material matches with the PLQE trend showing in Fig. 3B. We found that charge carrier recombination of perovskite in contact with tetracene is slower and more radiative compared to perovskite in contact with Spiro. The PLQE drops from 21 to 4% for perovskite-Spiro (by a factor of 5.2) compared to significantly lower decrease by a factor of 1.4 (to 14.8%) for perovskite-tetracene-Spiro configuration, indicating reduced interfacial nonradiative recombination and a cleaner interface in perovskite-tetracene (Fig. 3B). Under a lower-injection condition (lower fluences) where the PL decay is dominant through the nonradiative process rather than the extraction process, we observe a rapid decay followed by a long tail, indicating fast charge collection and longer lifetime for the charge carriers in perovskite-tetracene-Spiro compared to the quenched PL after the slower injection for the perovskite-Spiro (fig. S2F). These results collectively confirm lower nonradiative recombination and a cleaner interface in perovskite-tetracene.

Furthermore, we extracted the setup limited (time resolution of about 1.5 ns), initial PL signal (PL_*t* = 0_) that has a direct correlation with the charge extraction in the thin film (*36*). Notably, in the PL and PLQE results (Figs. 2C and 3B) where we use a continuum wavelength laser, the quenching mechanism is dominated by a nonradiative interface recombination because of the charge buildup by the carriers, which are extracted earlier. However, in the TRPL and initial PL (*t* = 0) measurements where a pulsed laser is used, the quenching in the initial PL value directly correlated to the charge extraction efficiency because of the lack of buildup charges in the pulsed measurement. We observe that the fastest hole extraction occurs in the triple layer of perovskite-tetracene-Spiro (fig. S2G). This confirms that tetracene-Spiro can act as efficient HTLs with a graded bandgap, leading to efficient extraction from the perovskite layer into the external circuit.

**Fig. 4:**
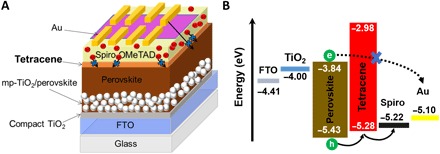
Solar cell architecture and energy diagram. (**A**) A device architecture schematic and (**B**) energy-level diagram obtained from UPS measurements (*30*) of a complete solar cell with graded doped HTLs of undoped tetracene with a doped Spiro layer. In the schematic, the red spheres are representative of Li^+^ ionic dopants present in doped Spiro layer, and the short and long arrows demonstrate the extrinsic ionic and Au migrations that are blocked by the undoped tetracene layer. The perovskite composition is Rb-passivated Cs_0.06_FA_0.79_MA_0.15_Pb(I_0.85_ Br_0.15_)_3_.

To validate our findings, we fabricated complete solar cells using the device architecture FTO/compact TiO_2_ (~30 nm)/thin mesoporous TiO_2_ (~200 nm)/perovskite (~500 nm)/tetracene (~120 nm)/Spiro-OMeTAD (~150 nm)/Au (80 nm), a device schematic of which is shown in Fig. 4A. In Fig. 4B, we show the respective energy levels of each layer as determined by ultraviolet photoelectron spectroscopy (UPS) (*30*) and the calculated bandgaps from the absorption spectra. As expected, the HOMO level of tetracene lies between the valence band of perovskite and the HOMO level of Spiro. This graded bandgap can facilitate effective hole injection from perovskite into the top electrode as we observe the fastest hole charge extraction from the triple layer of perovskite-tetracene-Spiro (fig. S2H). Furthermore, tetracene acts as an efficient electron-blocking layer because of the substantial difference between its lowest unoccupied molecular orbital and the conduction band of perovskite, a characteristic that could therefore diminish carrier recombination (*37*).

**Fig. 5:**
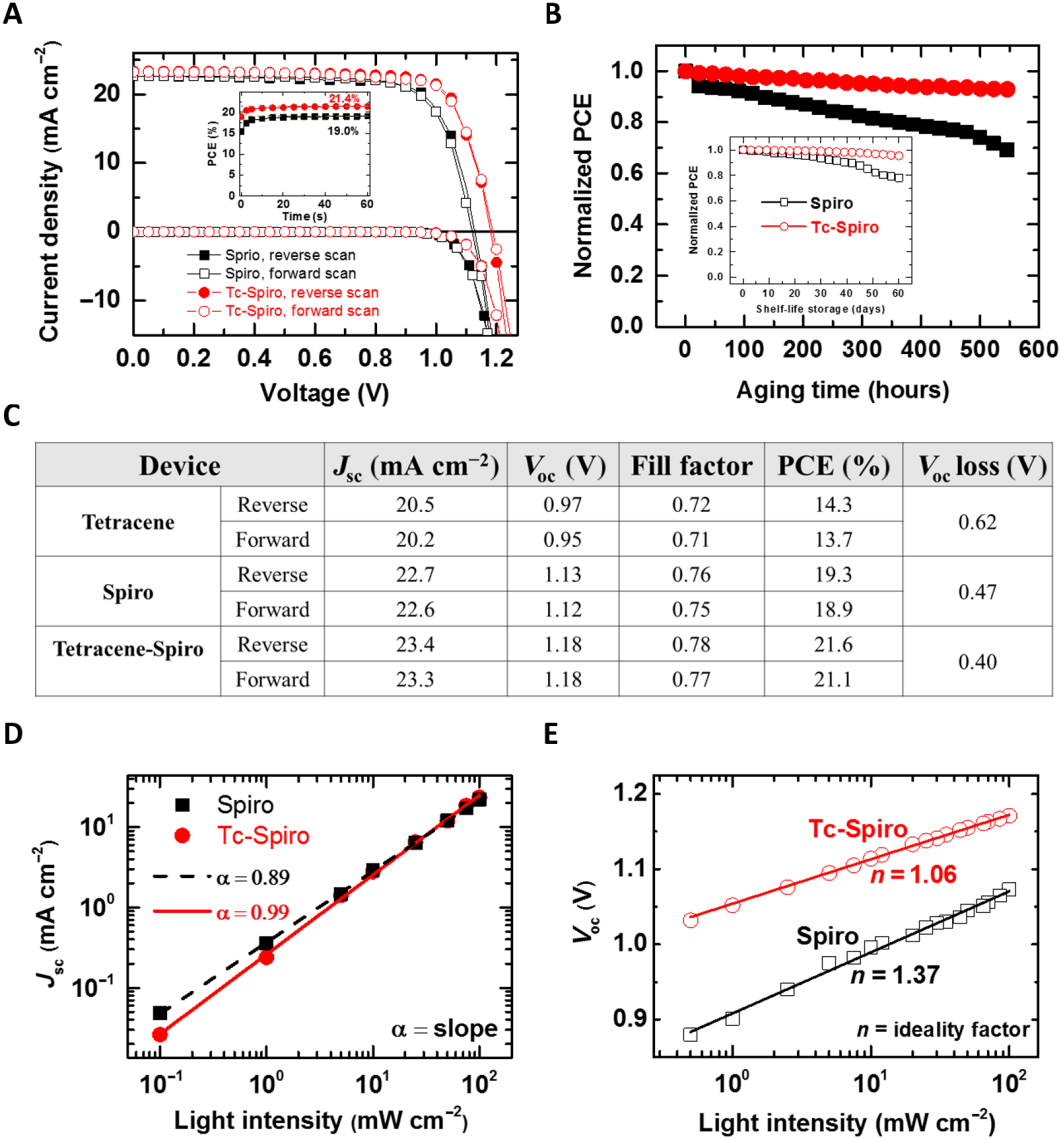
Enhanced PV performance and stability of solar cells. (**A**) Forward (open symbols) and reverse (closed symbols) *J-V* curves of champion solar cells with Spiro and tetracene-Spiro (Tc-Spiro) as HTL, measured under simulated solar illumination (AM1.5G, 100 mW cm^−2^) and dark conditions. Inset: Stabilized power output under the same conditions. (**B**) Stability curve of the solar cells at maximum power point under continuous AM1.5G illumination, N_2_ atmosphere, and stabilized temperature of 55°C. Inset: Shelf-life of devices stored in a nitrogen-filled glove box over 2 months and tested regularly under fully AM1.5G-simulated sunlight. The device stability at a higher temperature (i.e., 75°C) and under ambient conditions is presented in fig. S6. (**C**) PV parameters derived from *J-V* measurements of champion solar cells. The *V*_oc_ loss is the difference between the perovskite bandgap (1.59 eV, extracted from the external quantum efficiency onset) and measured *V*_oc_. (**D**) Short-circuit current (*J*_sc_) and (**E**) open-circuit voltage (*V*_oc_) Spiro and tetracene-Spiro devices plotted versus incident light intensity.

In Fig. 5C and fig. S3, we show device data for PSCs containing only tetracene as an HTL with an average PCE of 14.3%. To date, this is the highest PCE reported for a dopant-free linear acene derivative in PSCs. However, the average performance of these devices is lower than that of the Spiro-only solar cells (18.5% on average). This difference is primarily attributed to the poor ohmic contact between tetracene and Au, as observed extensively in organic field-effect transistors appearing as a high series resistance in the channel (*32*, *33*), leading to higher series resistance as it is evident from the lowest average fill factor for the tetracene-based PSCs (fig. S4C). To address this issue, we use a capping layer of doped Spiro to cover the tetracene layer and make a superior ohmic contact with the top electrode, providing a lower injection barrier. We then explored three different thicknesses of tetracene via thermal evaporation: 60, 120, and 240 nm. On the basis of the photovoltaic (PV) results shown in fig. S3 (A to C), we find that 120 nm of tetracene is the optimum thickness for achieving the maximum photocurrent and the highest enhancement in overall PCE.

In Fig. 5A, we show forward and reverse current-voltage (*J*-*V*) curves of champion devices for Spiro and tetracene-Spiro in the dark and under fully simulated sunlight (1 sun), with the extracted parameters given in Fig. 5C (see fig. S4 for device statistics). We find that device PCE, as calculated from current density-voltage (*J*-*V*) scans, increases from 14.3 to 21.6% when using the Spiro capping layer between tetracene and gold, with a maximum stabilized PCE of 21.4%. *J*-*V* hysteresis, commonly observed in PSCs, is negligible in our devices measured at different scan rates (fig. S5), confirming the high quality of the perovskite layer and its limited susceptibility for ion migration (*3*). The remarkable increase in open-circuit voltage (*V*_oc_) from Spiro to tetracene-Spiro devices (1.13 to 1.18 V) is consistent with the enhanced PLQE and reduced nonradiative recombination of perovskite when contacted with tetracene-Spiro (data in Fig. 3 and related discussion). In the Spiro-only devices, the interface between Spiro and perovskite acts as the main recombination center as apparent from the absorption in the perovskite-Spiro film below the perovskite bandgap arising from the presence of dopants in Spiro (Fig. 2B). These low-energy electron hole excitations lead to nonradiative recombination, resulting in a notable drop in the PLQE of perovskite (Fig. 3B) and therefore higher *V*_oc_ loss (~ 0.5 V). However, in the tetracene-Spiro solar cells, using tetracene as a nonquenching HTL in contact with perovskite and its combination with a capping layer of doped Spiro to retain a good ohmic contact with gold leads to the lowest *V*_oc_ loss (~0.4 V).

The increase in short-circuit current (*J*_sc_) for the tetracene-Spiro PSCs (from 22.7 to 23.4 mA cm^−1^) is also consistent with the increased hole extraction efficiency. Furthermore, the higher average fill factor in tetracene-Spiro devices can attributed to the more efficient hole transfer in these devices that leads to lower series resistance (≈65 ohm for tetracene-Spiro versus ≈110 ohm for Spiro-based devices) because of the presence of graded doping profile and graded HOMO level of tetracene and the HOMO level of Spiro (Fig. 4B). Briefly, the better PV performance of the PSCs with combined HTLs originates from a cleaner interface between perovskite and tetracene arising from the absence of dopants, as well as an effective ohmic contact between doped-Spiro and gold.

In fig. S3E, we show that *J*_sc_ values obtained from the *J*-*V* characteristics are well matched (within 4%) with the external quantum efficiency (EQE) obtained by the integration of the spectral response. In Fig. 5B, we conducted stability tests under maximum power conditions under simulated solar illumination (1000 W m^−2^). The ambient temperature during these tests was 55°C, and the atmosphere was dry N_2_. We observe a remarkable enhancement in the stability when using graded HTLs of tetracene and Spiro, with these devices retaining more than 90% of their initial performance after 550 hours of continuous operation. In contrast, only 68% of the initial PCE value was maintained for the Spiro-only devices. Furthermore, we found a negligible drop in shelf-life performance of the tetracene-Spiro device over 2 months; >95% of the initial PCE was retained. These stability figures are among the highest reported for PSCs (*12*, *20*). The substantial enhancement in the stability of tetracene-Spiro–based PSCs likely originates from the clean interface between tetracene and perovskite and from the presence of tetracene acting as a barrier layer toward the migration of dopants from Spiro-OMeTAD and metal diffusion from the Au electrode into the perovskite (Fig. 4A). To further confirm the latter claim, we performed the stability tests at a higher temperature (i.e., 75°C) at the maximum power point that is reported to be a strong enabler for extrinsic ionic migration from the Spiro doped with lithium salt and for metal migration (*25*, *26*). Expectedly, the PCE of Spiro-only solar cells dropped catastrophically during continuous operation at an elevated temperature. On the contrary, using graded doped HTLs in PSCs significantly enhanced their operational stability, retaining more than 90% of its initial stability after 300 hours (fig. S6).

To probe the impact of graded HTLs on charge recombination in the PSC, we performed light intensity–dependent PV characterization on the Spiro and tetracene-Spiro devices (see *J*-*V* curves in fig. S7). In Fig. 5C, we demonstrate the power law dependence of the *J*_sc_ on incident light intensity, where tetracene-Spiro solar cells exhibit α = 0.99 compared to α = 0.89 for Spiro-only devices, suggesting enhanced charge extraction in the former (*38*). Furthermore, the larger slope (α) indicates that more facile trap filling in the devices and therefore a higher rate of increase in the current density for the tetracene-Spiro devices suggest lower trap density compared to the Spiro-based solar cells. In Fig. 5D, we show the *V*_oc_ as a function of incident light intensity on a linear log scale where the ideality factor (*n*) calculated from the slope is representative of the charge carrier recombination process (*38*). It is noted that the ideality factor *n* approaches unity for bimolecular charge carrier recombination, while *n* approaches 2 for Shockley Reed Hall (SRH) trap-assisted recombination (*39*). We found that the ideality factor for tetracene-Spiro devices approaches *n* = 1.06, pointing toward the bimolecular type of carrier recombination. This shows that the carriers in the tetracene-Spiro devices recombine primarily via a band-to-band radiative process where traps can be passivated within thermal equilibrium condition, enabling the tetracene-Spiro devices to reach higher PCE and minimal *V*_oc_ losses (Fig. 5C). However, for Spiro-only devices, the ideality factor is 1.37, resulting in an SRH-type trap-assisted charge carrier recombination process primarily at the charge collection interfaces. This can further confirm the cleaner interfaces between perovskite and the HTL (i.e., tetracene) in tetracene-Spiro devices. In addition, suppression of SRH-type recombination in tetracene-Spiro devices reflected in the substantial enhancement of the corresponding average fill factor (*1*) (fig. S4C).

**Fig. 6:**
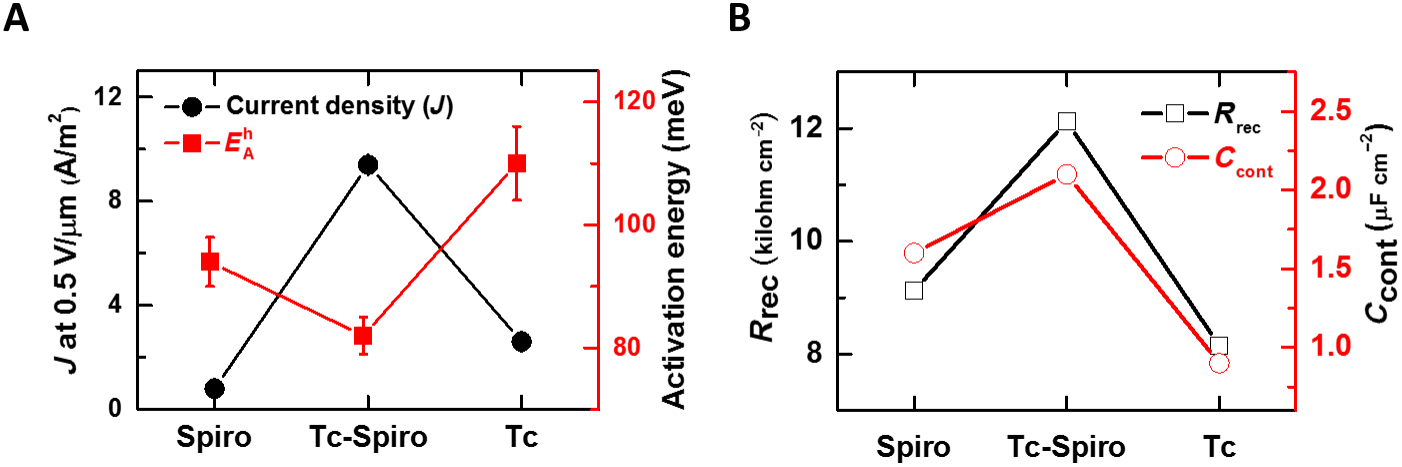
Enhanced hole transport in PV devices. (**A**) The trends in current density (*J*) measured at a field of 0.5 V/μm and activation energy (*E*^h^_SCL_) for Spiro-, tetracene-Spiro–, and Tc-based devices extracted from temperature-dependent *J*-*V* characteristics of hole-only devices (FTO/perovskite/HTL/Au) as shown in fig. S8. (**B**) The evolution for recombination resistance (*R*_rec_) and contact capacitance (*C*_cont_) extracted from the electrochemical impedance spectroscopy measurements on the complete solar cells consisting of different HTLs (see fig. S9).

To investigate the hole transport behavior in a different configuration of HTLs, we fabricated hole-only (FTO/perovskite/HTL/Au) devices for space-charge–limited current (SCLC) measurement. In Fig. 6A, we show the plot of the current density (*J*) at 0.3 V where the transport is expected to be hole injection limited. We observed a remarkable enhancement in the current density for tetracene-Spiro devices compared to Spiro-only devices. Furthermore, we estimated the activation energy for hole transport (*E*^h^_A_) from temperature-dependent *J*-*V* measurements (fig. S8). The activation energy is then estimated from a typical Arrhenius fits to current density in the *J* ∝ *V*^2^ regime. In this device geometry, the magnitude of current density has a contribution from the bulk conductivity of the perovskite layer and the effective injection barriers at the electrodes. Considering the fact that only the HTL on top of the perovskite film is varied in these devices, this variation in the current density and activation energy of these hole-only devices could be attributed to the decreased injection barrier for hole transport due to the graded energy levels of tetracene and Spiro as the HTL. The observed enhancement in the current density and the decrease in *E*^h^_A_ are consistent with the enhancement in the *J*_sc_ and decrease in the recombination, which could boost the *V*_oc_, resulting in an overall improvement in the PCE.

To understand further the transport mechanism, we performed electrochemical impedance spectroscopy (EIS) on PSC fabricated with different HTLs. We performed the EIS measurement on the complete solar cells operating under 1-sun illumination and bias conditions close to the open-circuit conditions (fig. S9, A to C). The EIS spectra were fitted with an appropriate equivalent circuit to estimate parameters related to the charge recombination and polarization relaxation of the perovskite layers (Fig. 6B and fig. S9). After fitting the equivalent circuit to the EIS data, we found the recombination resistance (*R*_rec_) to be maximum for tetracene-Spiro, which is consistent with the trend of higher *J*_sc_ magnitude (Fig. 5C). Furthermore, we observe an enhancement in the contact capacitance (*C*_cont_) of the complete tetracene-Spiro devices over Spiro-only devices, which is consistent with the *V*_oc_ trend (Fig. 5C).

## DISCUSSION

In summary, we have demonstrated that thermally evaporated, dopant-free tetracene can act as an efficient HTL in PSCs while maintaining a high luminescence yield of perovskite in the device architecture. However, because of the poor ohmic contact between tetracene and the metal electrode, we found that depositing a capping layer of doped Spiro-OMeTAD not only is beneficial to make a superior interface with top contact but also facilitates the hole injection from perovskite into the external circuit via graded energy levels. We performed a series of measurements including pulsed PL, TRPL, and external PLQE to confirm faster hole injection of perovskite to HTL, as well as slower and more radiative charge carrier recombination of the perovskite when interfaced with tetracene-Spiro (e.g., external PL yield of 15% for perovskite-tetracene-Spiro compared to 5% for perovskite-Spiro). These results collectively show the beneficial effects of an energy-level cascade at a charge collection electrode and of having a graded doping profile with the combined HTLs, providing a clean interface between perovskite and tetracene and minimizing the interfacial nonradiative recombination losses. We further validate the applicability of our findings by fabricating complete solar cells with PCE exceeding 21%. Open-circuit voltages in the efficient devices reach 1.18 V, indicating very low voltage losses (0.40 V). Last, the stability tests under continuous illumination show outstanding improvement in the stability of the tetracene-Spiro devices, maintaining more than 90% of the initial stability after 550 hours. Our study paves the way toward minimization of interfacial losses and stabilization of high-efficiency PSCs using cascaded high-mobility molecular semiconductors as suitable hole extraction layers with a graded doping profile.

## MATERIALS AND METHODS

### Film and device fabrication

All the organic cation salts, the lead compounds, and RbI and CsI were purchased from Dyesol, TCI Chemicals, and Alfa Aesar, respectively. Spiro-OMeTAD was purchased from Borun Chemicals and used as received. Unless otherwise stated, all other materials were purchased from Sigma-Aldrich.

The rubidium-doped triple cation–based perovskite [e.g., Rb-doped Cs_0.06_FA_0.79_MA_0.15_Pb(I_0.85_ Br_0.15_)_3_] was prepared by dissolving PbI_2_ (1.2 M), FAI (1.11 M), MABr (0.21 M), and PbBr_2_ (0.21 M) in a mixture of anhydrous *N*,*N*′-dimethylformamide/dimethyl sulfoxide (DMF/DMSO) (4:1, volume ratios), followed by addition of 5% (v/v) from CsI stock solution (1.5 M in DMSO) and RbI stock solution (1.5 M in DMF/DMSO; 4:1, volume ratios), respectively. We then spin-coated the perovskite solutions using a two-step program at 2000 and 6000 rpm for 10 and 40 s, respectively, and dripping 150 μl of chlorobenzene after 30 s. We then annealed the films at 100°C for 1 hour. All the film preparations were performed in a nitrogen-filled glove box. The devices were fabricated following the same procedures for substrate preparation and deposition of both electron and HTLs (i.e., TiO_2_ and Spiro-OMeTAD) as in our previous work (*7*). The tetracene layer was deposited via thermal evaporation on the perovskite layer with the evaporation rate of 0.2 nm s^−1^ and chamber pressure of 10^−7^ mbar.

### Scanning electron microscopy

The surface morphology of the films was examined using a field-emission SEM (Merlin). An electron beam accelerated to 3 kV was used with an in-lens detector. For cross-sectional scanning electron microscopy, the samples were fabricated by mechanical cutting using a diamond pen and imaged in a high-resolution scanning microscope (S-5500, Hitachi).

### Steady-state absorption and PL characterization

Absorption spectra were recorded with a PerkinElmer LAMBDA 1050 spectrophotometer equipped with an integrating sphere to account for reflected and transmitted light. PDS measurements were acquired on a custom-built setup by monitoring the deflection of a fixed wavelength (670 nm) laser probe beam following absorption of each monochromatic pump wavelength by a thin film immersed in an inert liquid FC-72 Fluorinert (3M Company). PL quantum yield measurements were taken by mounting perovskite films or encapsulated device stacks in an integrating sphere and photoexciting with a 532-nm continuous-wave laser. The laser and the emission signals were measured and quantified using a calibrated Andor iDus DU490A InGaAs detector for the determination of PLQE.

### Time-resolved PL

TRPL measurements were acquired with a gated intensified charge-coupled device (iCCD) camera system (iStar DH740 CCI-010, Andor Technology) connected to a grating spectrometer (SR303i, Andor Technology). Excitation was performed with femtosecond laser pulses that were generated in a home-built setup by second harmonic generation in a BaB_2_O_4_ crystal from the fundamental output (pulse energy, 1.55 eV; pulse length, 80 fs) of a Ti:Sapphire laser system (Solstice, Spectra-Physics). Temporal resolution of the PL emission was obtained by measuring the PL from the sample by stepping the iCCD gate delay relative to the pump pulse. The gate width was 20 ns.

### Ultraviolet photoelectron spectroscopy

The UPS system operates by emitting photons of a fixed energy of 21.2 eV (58.4 nm) via a helium gas discharge lamp. On the basis of Einstein’s photoelectric law, photoelectrons are able to escape from the surface of a sample if their kinetic energy is sufficient to overcome the sum of the binding energy of their initial level (taken with reference to *E*_F_) and the material’s work function Φ = *E*_VAC_ − *E*_F_. Here, the secondary electron cutoff represents electrons without any kinetic energy. Consequently, a material’s Fermi-level position with respect to the vacuum level (its work function) can be computed by determining the secondary electron cutoff from a UPS spectrum and subtracting it from the incident photon energy adjusted for any external potential applied during the measurement.

### Solar cell characterization

Current-voltage characteristics were recorded by applying an external potential bias to the cell while recording the generated photocurrent with a digital source meter (model 2400, Keithley Instruments). The light source was a 450-W xenon lamp (Oriel) equipped with a Schott K113 Tempax sunlight filter (Praezisions Glas & Optik GmbH) to match the emission spectrum of the lamp to the AM1.5G standard. Before each measurement, the exact light intensity was determined using a calibrated Si reference diode equipped with an infrared cutoff filter (KG-3, Schott). EQE spectra were recorded as a function of wavelength under a constant white-light bias of approximately 5 mW cm^−2^ supplied by an array of white light–emitting diodes. The excitation beam coming from a 300-W xenon lamp (ILC Technology) was focused through a Gemini 180 double monochromator (Jobin Yvon Ltd.) and chopped at approximately 2 Hz. The signal was recorded using a Model SR830 DSP Lock-In Amplifier (Stanford Research Systems). All measurements were conducted using a nonreflective metal aperture of 0.105 cm^2^ to define the active area of the device and avoid light scattering through the sides.

For stability measurements, the solar cells were transferred to a sealable device holder under nitrogen-filled glove box conditions. During testing, the device holder was continuously purged with dry nitrogen, prefiltered (Super Clean, Scientific Glass Technology) to minimize residual oxygen, moisture, and hydrocarbon content. A Newport solar simulator with equivalent AM1.5G 1-sun output was used to illuminate the entire device substrate; short wavelengths were filtered using a 435-nm long-pass filter (FGL435, Thorlabs). Aging under these conditions resulted in a chamber ambient temperature of approximately 55°C, as measured by a thermistor next to the solar cell device, which was reached within 30 min of the experiment commencing. Photocurrent characteristics were recorded by holding close to the maximum power point voltage (as ascertained from an initial *J*-*V* curve) using a Keithley 2636 System SourceMeter (SMU) and a custom-written LabVIEW virtual instrument code. Devices were stored in a nitrogen-filled glove box in the dark between shelf-life measurements.

### Bulk transport measurements

Hole-only devices were fabricated on precleaned FTO substrates (15 ohm sq^−1^). The mesoporous perovskite layer with the different interfacial layers was fabricated in the same way as described for the solar cell device fabrication. Metal electrodes Au for the devices were coated by thermal evaporation (10^−6^ mbar, 0.1 A^0^/s, 80 nm thick). The devices were characterized using a Keithley 4200-SCS Semiconductor Characterization System and temperature was varied using a radio frequency probe station from Cryo Technologies.

### Electrochemical impedance spectroscopy

EIS of the solar cells was carried out using a precision impedance analyzer HP4294A, while the device was illuminated using a home-built light source with close to 1-sun intensity. The device was driven by a small ac frequency of 30 mV, and obtained impedance parameters were fitted with a standard circuit (see fig. S9) to estimate the physical parameters. In the EIS measurements (using the equivalent circuit in fig. S9C), the device is driven at a small ac voltage of 30 mV, while the frequency is swept from 100 Hz to 10 MHz. In hybrid semiconductors such as perovskites, charge transport has a contribution from both the electronic and ionic contributions. Considering these, the equivalent circuit has a combination of resistor-capacitor circuits that corresponds to both the low- and high-frequency components. The components in the EIS circuit can be described as *R*_s_ (series resistance), *R*_rec_ (recombination resistance), *R*_dr_ (resistance due to dielectric Debye relaxation), and *C*_dr_ (capacitance due to Debye relaxation). It is important to understand the origin of these components in the abovementioned equivalent circuit. The series resistance (*R*_s_) originates from the resistance of the FTO substrates used for device fabrication (13 ohm sq^−1^). *R*_rec_ and *C*_cont_ are parts of the high-frequency impedance component, which corresponds to the impedance originating from different interface layers and is also related to the overall electronic charge transport properties of the perovskite layer. The components *R*_dr_ and *C*_dr_ correspond to the low-frequency component, which mainly originates from the Debye ionic relaxation of the perovskite layer. It should be noted that we have chosen a series combination of the low-frequency component instead of the parallel combination because of the fact that the properties such as open-circuit voltage in perovskite semiconductors are observed to be dependent on the capacitance originating from ionic layer or the chemical capacitance of the perovskite layer. In fig. S10, we performed the capacitance measurement on the solar cells using 4200-SCS for all the devices under dark conditions using an ac voltage of 30 mV, and the frequency swept across 1 kHz to 10 MHz. Note that these measurements were performed under dark conditions without any bias conditions to understand the inherent effect of the double HTL on the capacitive behavior of the solar cell. Nevertheless, the measurement is indicative of the expected trend in the devices.

## Supplementary Material

http://advances.sciencemag.org/cgi/content/full/5/2/eaav2012/DC1

## SUPPLEMENTARY MATERIALS

Supplementary material for this article is available at http://advances.sciencemag.org/cgi/content/full/5/2/eaav2012/DC1 Fig. S1. Morphological and cross-sectional characterization of graded doped HTLs. Fig. S2. Optical and magnetic field characterization of the perovskite thin films interfaced with a different configuration of HTLs. Fig. S3. PV characterization of PSCs with different thicknesses of tetracene in graded doped HTLs configuration. Fig. S4. Device statistics. Fig. S5. Hysteresis behavior of graded doped HTL-based PSCs at different scan rates. Fig. S6. Device stability. Fig. S7. The current-voltage characteristic as a function of light intensity for PSCs with different HTL configurations. Fig. S8. Temperature-dependent SCLC charge transport characterization of hole-only PSCs. Fig. S9. EIS of PSCs with different HTL configurations. Fig. S10. Typical capacitive response of the perovskite layers interfaced with different HTLs.
