# Supplementary material for: Charge extraction via graded doping of hole transport layers gives highly luminescent and stable metal halide perovskite devices
Source: Sci Adv. 2019 Feb 15;5(2):eaav2012. doi: 10.1126/sciadv.aav2012 (PMC6377269; doi:10.1126/sciadv.aav2012)
Supplement: http://advances.sciencemag.org/cgi/content/full/5/2/eaav2012/DC1 [file supp_5_2_eaav2012__index.html]

Science Advances | Science Advances

## Supplementary Materials

**This PDF file includes:**

- Fig. S1. Morphological and cross-sectional characterization of graded doped HTLs.
- Fig. S2. Optical and magnetic field characterization of the perovskite thin films interfaced with a different configuration of HTLs.
- Fig. S3. PV characterization of PSCs with different thicknesses of tetracene in graded doped HTLs configuration.
- Fig. S4. Device statistics.
- Fig. S5. Hysteresis behavior of graded doped HTL-based PSCs at different scan rates.
- Fig. S6. Device stability.
- Fig. S7. The current-voltage characteristic as a function of light intensity for PSCs with different HTL configurations.
- Fig. S8. Temperature-dependent SCLC charge transport characterization of hole-only PSCs.
- Fig. S9. EIS of PSCs with different HTL configurations.
- Fig. S10. Typical capacitive response of the perovskite layers interfaced with different HTLs.

Download PDF

**Files in this Data Supplement:**

- Adobe PDF - aav2012\_SM.pdf
