# Supplementary material for: Charge extraction via graded doping of hole transport layers gives highly luminescent and stable metal halide perovskite devices
Source: Sci Adv. 2019 Feb 15;5(2):eaav2012. doi: 10.1126/sciadv.aav2012 (PMC6377269; doi:10.1126/sciadv.aav2012)
Supplement: http://advances.sciencemag.org/cgi/content/full/5/2/eaav2012/DC1 [file aav2012_SM.pdf]

Science Advances
AAAS

advances.sciencemag.org/cgi/content/full/5/2/eaav2012/DC1

Supplementary Materials for

Charge extraction via graded doping of hole transport layers gives highly luminescent and stable metal halide perovskite devices

Mojtaba Abdi-Jalebi*, M. Ibrahim Dar, Satyaprasad P. Senanayak, Aditya Sadhanala, Zahra Andaji-Garmaroudi, Luis M. Pazos-Outón, Johannes M. Richter, Andrew J. Pearson, Henning Sirringhaus, Michael Grätzel, Richard H. Friend*

*Corresponding author. Email: ma571@cam.ac.uk (M.A.-J.); rhf10@cam.ac.uk (R.H.F.)

Published 15 February 2019, *Sci. Adv.* **5**, eaav2012 (2019)
DOI: 10.1126/sciadv.aav2012

This PDF file includes:

Fig. S1. Morphological and cross-sectional characterization of graded doped HTLs.
Fig. S2. Optical and magnetic field characterization of the perovskite thin films interfaced with a different configuration of HTLs.
Fig. S3. PV characterization of PSCs with different thicknesses of tetracene in graded doped HTLs configuration.
Fig. S4. Device statistics.
Fig. S5. Hysteresis behavior of graded doped HTL-based PSCs at different scan rates.
Fig. S6. Device stability.
Fig. S7. The current-voltage characteristic as a function of light intensity for PSCs with different HTL configurations.
Fig. S8. Temperature-dependent SCLC charge transport characterization of hole-only PSCs.
Fig. S9. EIS of PSCs with different HTL configurations.
Fig. S10. Typical capacitive response of the perovskite layers interfaced with different HTLs.

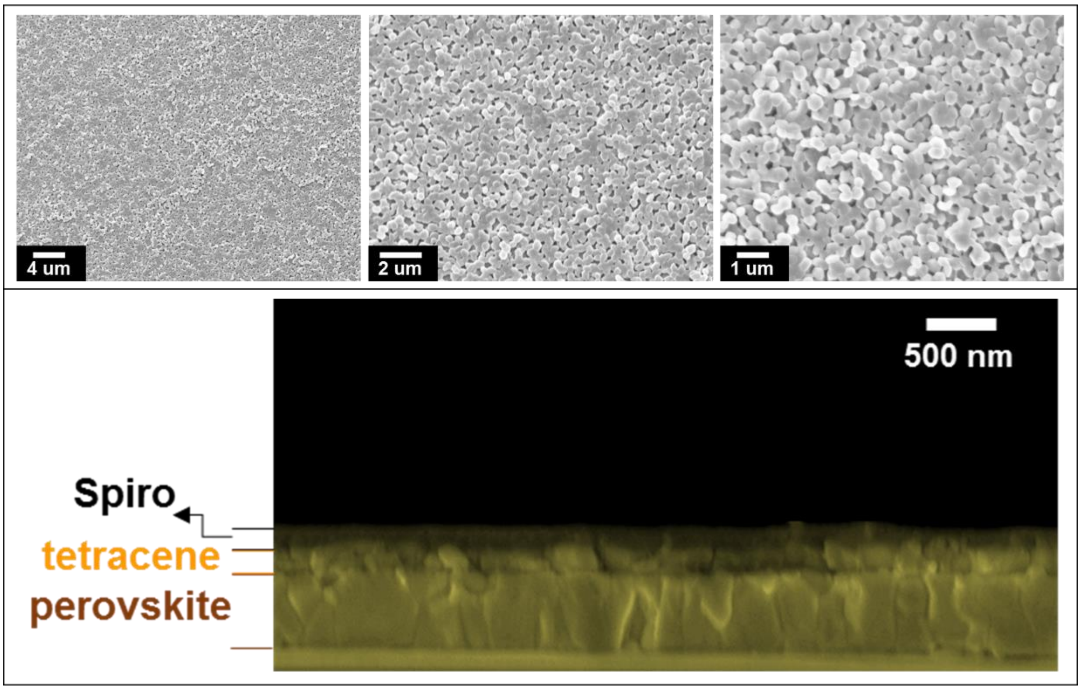

Fig. S1. Morphological and cross-sectional characterization of graded doped HTLs. (top panel) Top view SEM images of perovskite/tetracene thin films washed with chlorobenzene solvent using the same deposition condition as Spiro top layer in different magnifications. (bottom panel) Cross-sectional SEM images of perovskite/tetracene/Spiro multilayer films deposited on glass.

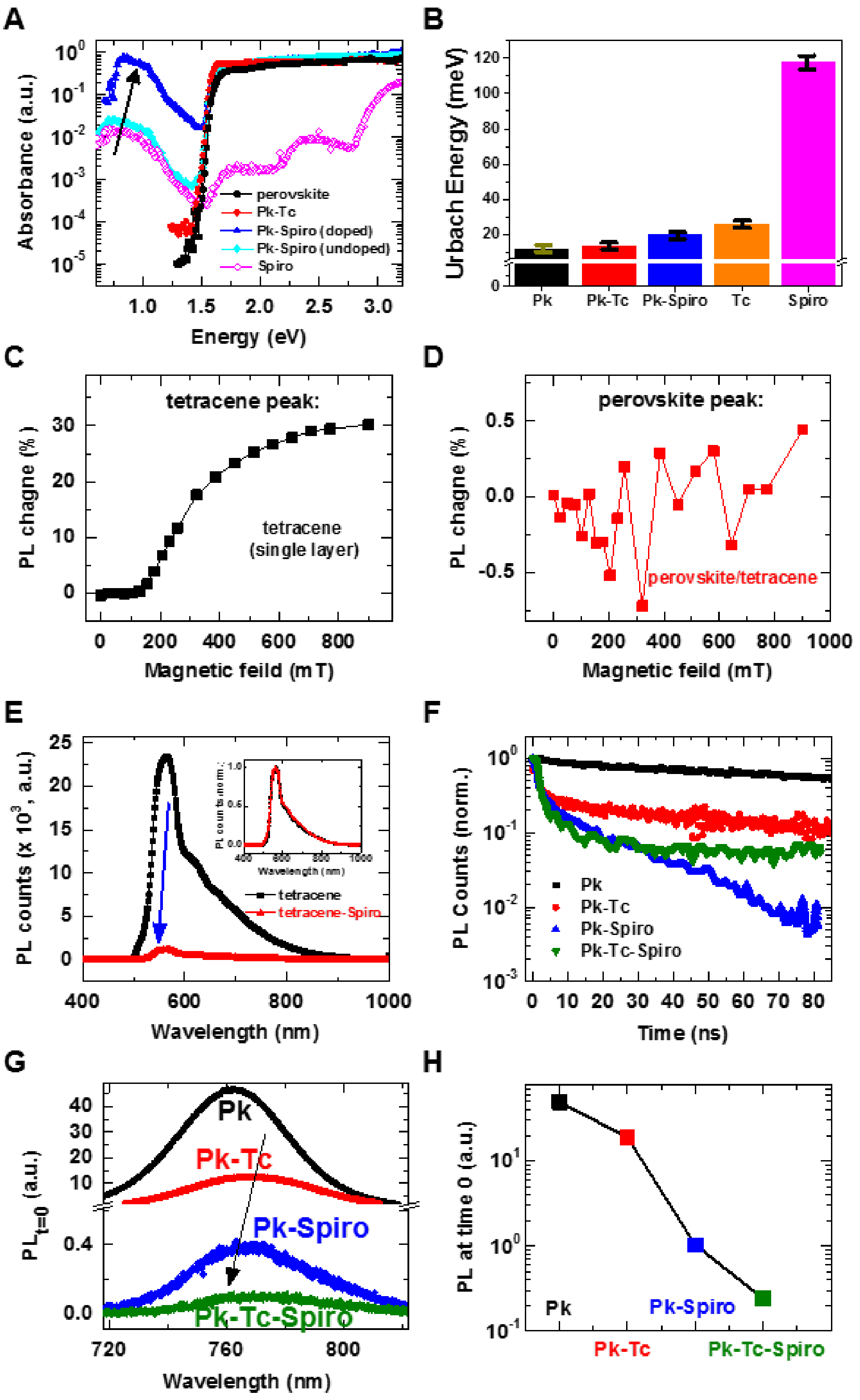

Fig. S2. Optical and magnetic field characterization of the perovskite thin films interfaced with a different configuration of HTLs. (**A**) PDS absorption spectra of perovskite, perovskite/doped-Spiro, perovskite/un-doped Spiro and Spiro thin films. (**B**) The

Urbach energies calculated from the corresponding PDS absorption spectra. Magnetic field effect on fluorescence of (**C**) tetracene PL peak at 530 nm and (**D**) perovskite peak at 760 nm for bilayer of perovskite-tetracene. The magnetic PL response was recorded upon illumination of 405nm continuous laser excitation from tetracene side.

(**E**) Photoluminescence spectra, excitation at 405 nm, of the tetracene and tetracene-Spiro layers. Inset: Normalized PL spectra for these films. (**F**) Time-resolved PL decays of the films with excitation at 407 nm and pulse fluence of 0.01 $\mu J.cm^{-2}$. (**G**) Photoluminescence spectra and (**H**) the initial PL values at 1.5 ns after excitation at 400 nm with a 100 fs laser pulse. Note that Pk and Tc refer to perovskite and tetracene, respectively.

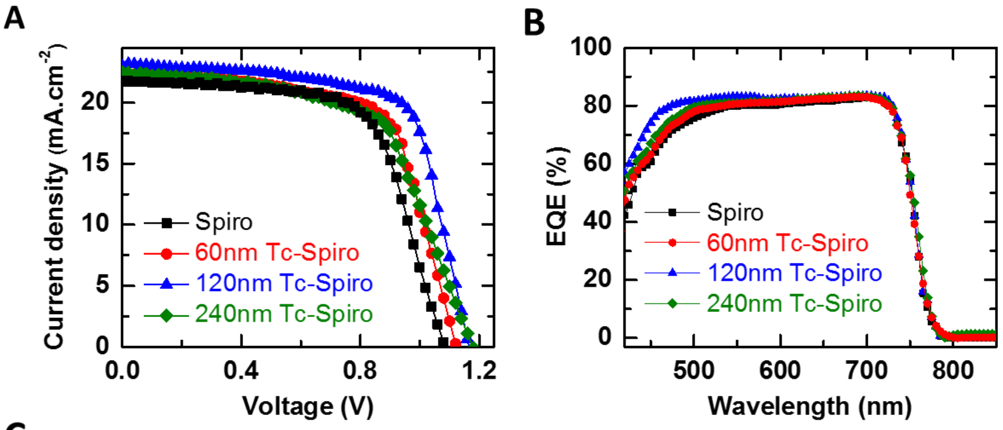

C

Device	J_{sc} [mA.cm^{-2}]	V_{oc} [V]	Fill factor	PCE [%]
**Spiro**	21.8	1.080	0.65	15.36
**60 nm Tc-Spiro**	22.4	1.122	0.66	16.68
**120 nm Tc-Spiro**	23.0	1.165	0.70	18.76
**240 nm Tc-Spiro**	22.5	1.176	0.61	16.12

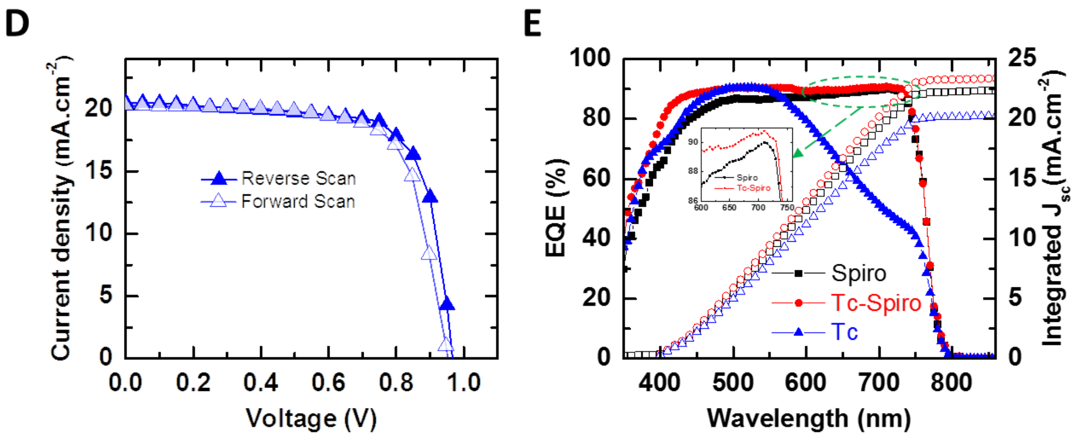

Fig. S3. PV characterization of PSCs with different thicknesses of tetracene in graded doped HTLs configuration. (**A**) *J-V* characteristics and (**B**) external quantum efficiencies (EQE) of solar cells with a different thickness of thermally evaporated tetracene interlayer, measured under simulated solar illumination (AM1.5, 100 mW.cm^{-2}). (**C**) Device parameters for different thickness of thermally evaporated tetracene interlayer measured under full simulated solar illumination conditions (AM1.5, 100 mW.cm^{-2}). (**D**) Forward (open symbols) and reverse (closed symbols) *J-V* curves of champion solar cells with tetracene as HTL, measured under simulated solar illumination (M1.5, 100 mW.cm^{-2}).

(**E**) External quantum efficiencies (EQE) and integrated short-circuit current for Spiro,

Tc-Spiro and Tc based solar cells. The drop in the EQE of the Tc only devices refers to the poor ohmic contact between tetracene and gold limiting the hole-injection.

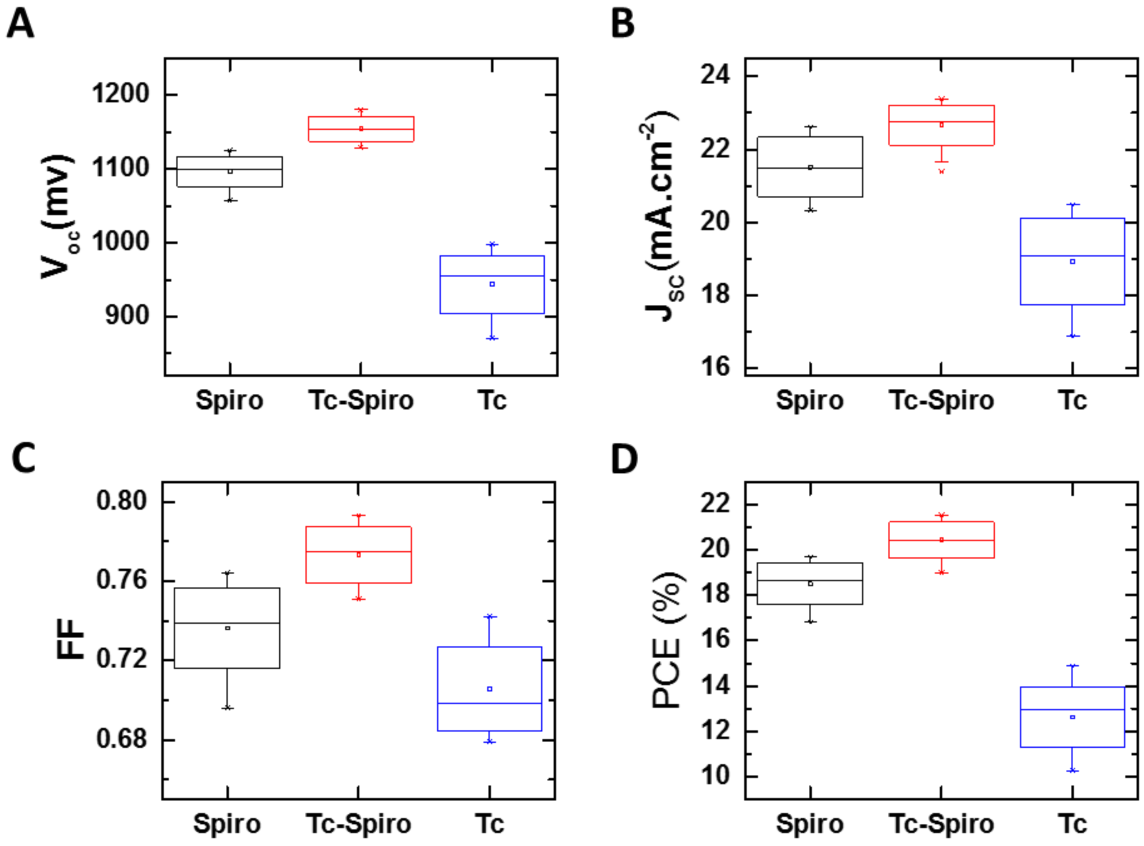

Fig. S4. Device statistics. (**A-D**) Box and whisker plots to summarise the statistics of photovoltaic parameters of Spiro, Tc-Spiro and Tc based perovskite solar cells, 15 of each, were measured under full-simulated solar illumination conditions (AM1.5, 100 mW.cm^{-2}) and scanned at a rate of 15 mV/s. The boxes represent the interquartile range, with the median represented by the line dividing the boxes, and the whiskers are determined by the 5th and 95th percentiles. The mean is given by the open square symbols, and the starred symbols represent the maximum and minimum values. Note that Tc refers to tetracene.

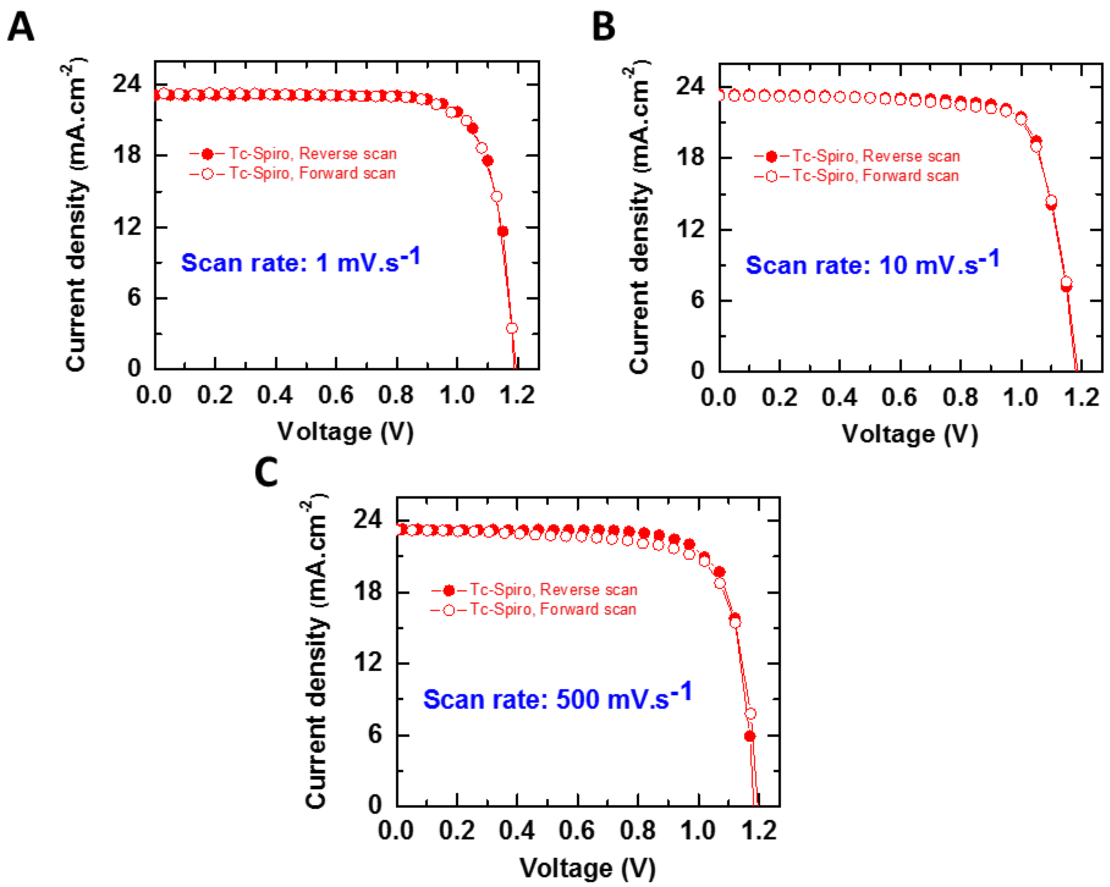

Fig. S5. Hysteresis behavior of graded doped HTL-based PSCs at different scan rates.

Current-voltage characteristic of tetracene-Spiro devices at different scan rates of (**A**) 1 mV.S^{-1}, (**B**) 10 mV.S^{-1} and (**C**) 500 mV.S^{-1}. Note that Tc refers to tetracene.

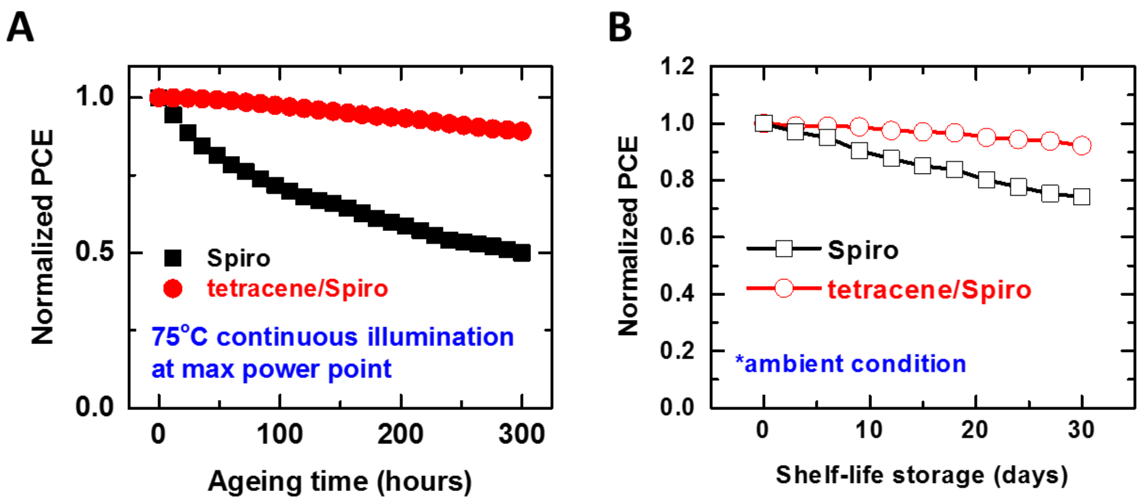

Fig. S6. Device stability. (**A**) Stability curve of the solar cells at maximum power point under continuous AM 1.5 G illumination, N_2 atmosphere and stabilised temperature of 75 ^{o}C. (**B**) Shelf-life of devices stored in ambient condition over a month and tested regularly under full AM1.5 simulated sunlight.

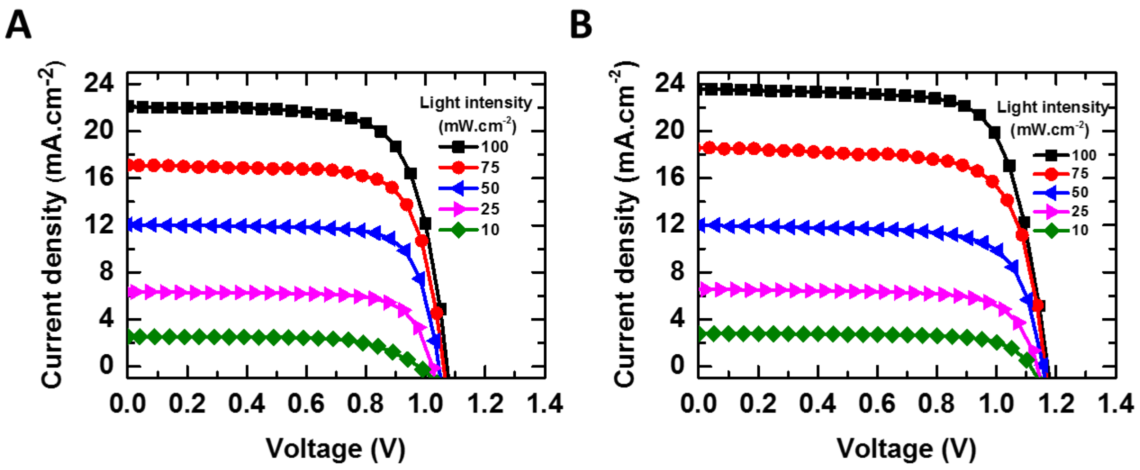

Fig. S7. The current-voltage characteristic as a function of light intensity for PSCs with different HTL configurations. J-V characteristics of solar cells with (**A**) Spiro and (**B**) Tc-Spiro HTLs under different light intensities.

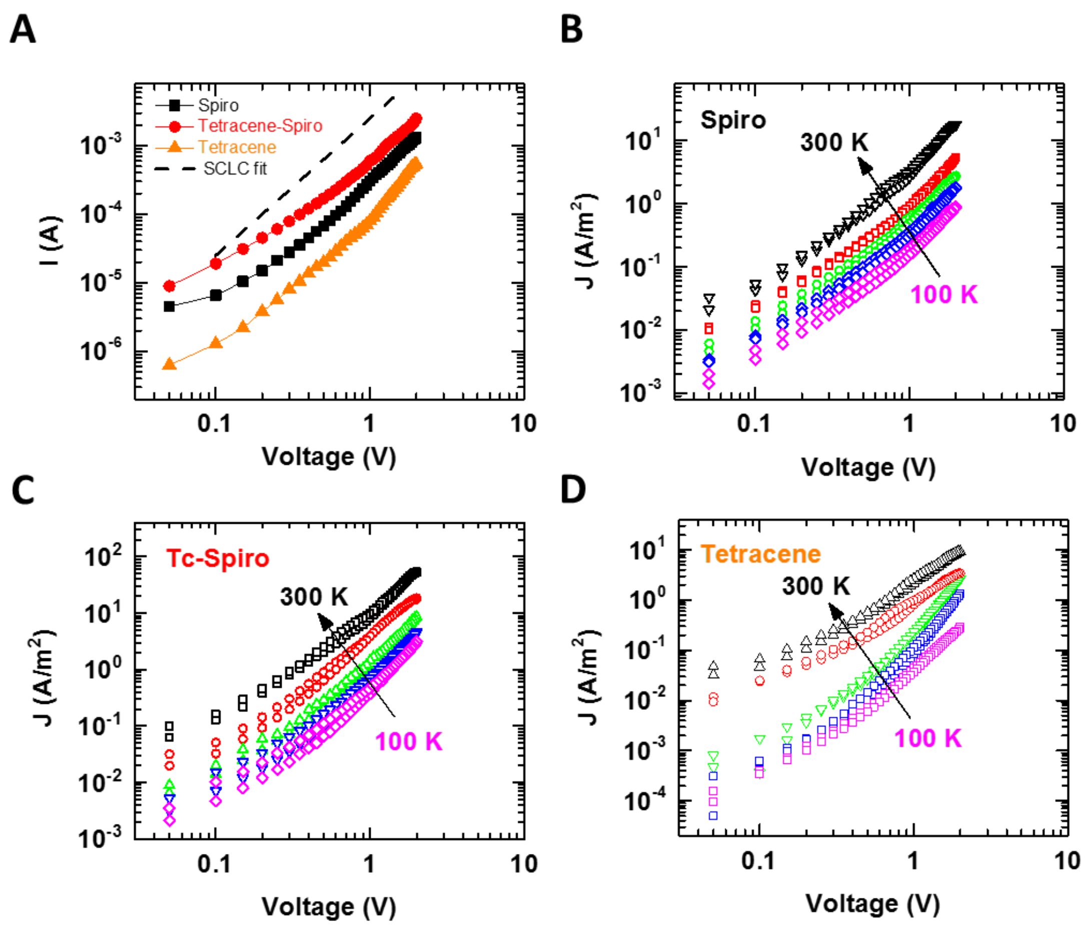

Fig. S8. Temperature-dependent SCLC charge transport characterization of hole-only PSCs. (**A**) I-V characteristics of hole-only perovskite devices with different HTL configurations measured at room temperature. I-V characteristics of (**B**) Spiro, (**C**) Tc-Spiro and (**D**) tetracene hole only devices (FTO/perovskite/HTL/Au) at different temperatures, utilized for estimating the space charge limited activation energy for hole transport. The temperature gradient between each curve is 50 K.

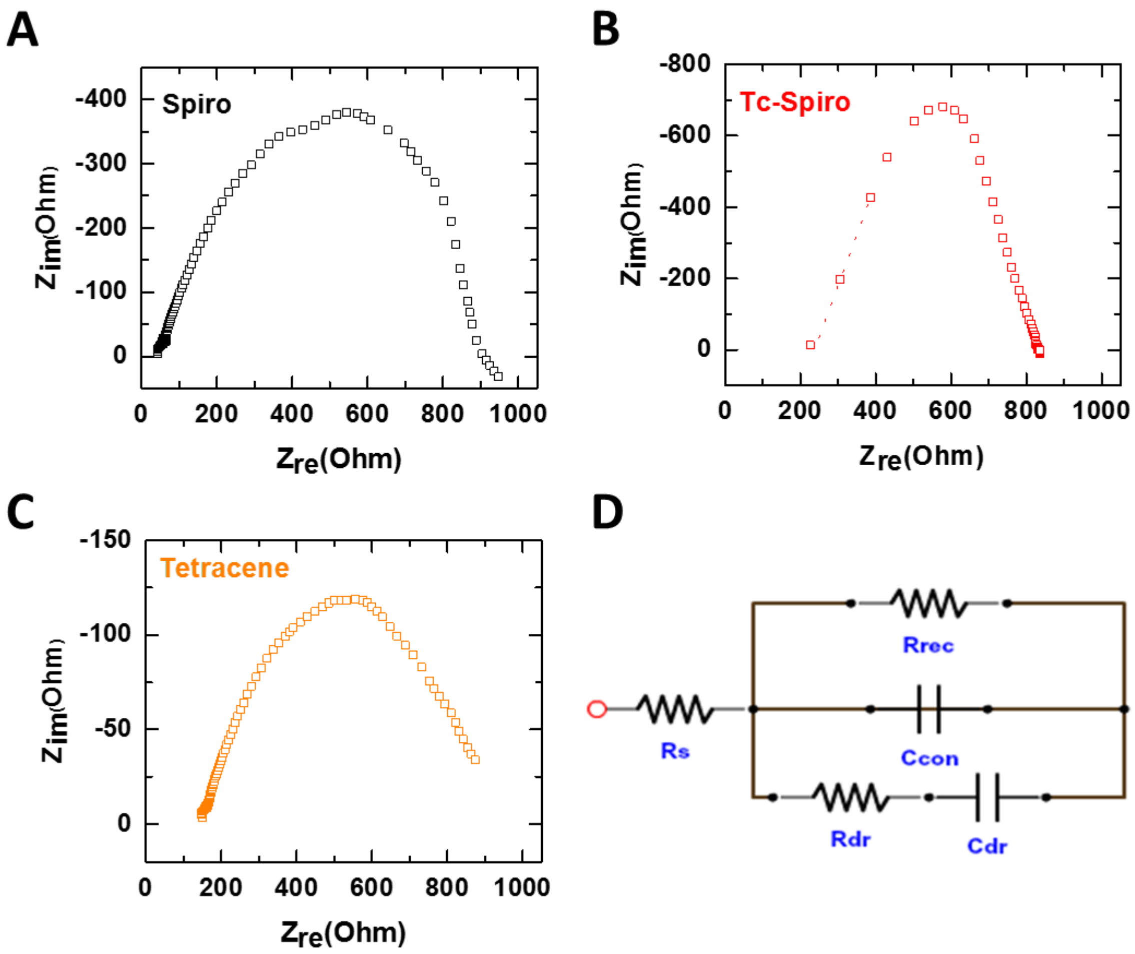

Fig. S9. EIS of PSCs with different HTL configurations. Typical EIS spectra measured on the perovskite solar cells with (**A**) Spiro, (**B**) Tc-Spiro and (**C**) tetracene as HTL.

(**D**) Equivalent circuit for electrochemical impedance spectroscopy (EIS) measurement.

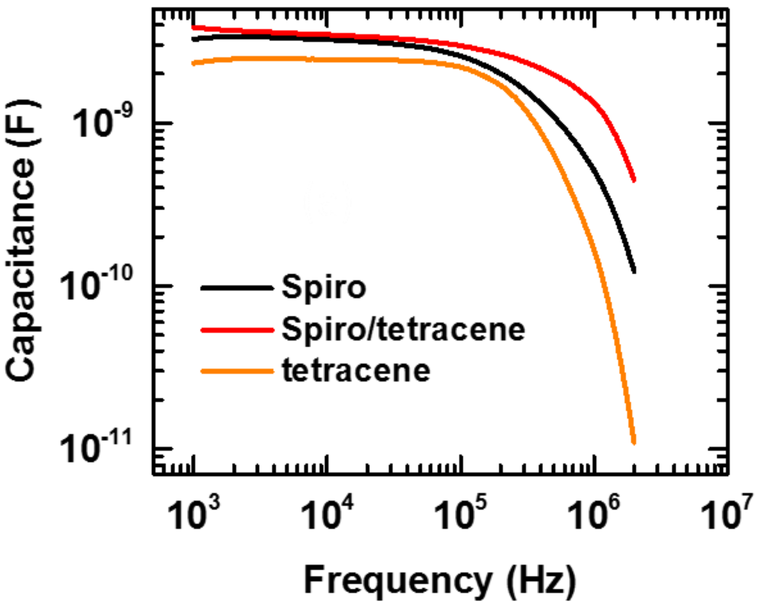

Fig. S10. Typical capacitive response of the perovskite layers interfaced with different HTLs.
